# Developing cookies formulated with goat cream enriched with conjugated linoleic acid

**DOI:** 10.1371/journal.pone.0212534

**Published:** 2019-09-23

**Authors:** Ana C. S. Costa, Diego E. Pereira, Caio M. Veríssimo, Marcos A. D. Bomfim, Rita C. R. E. Queiroga, Marta S. Madruga, Susana Alves, Rui J. B. Bessa, Maria E. G. Oliveira, Juliana K. B. Soares

**Affiliations:** 1 Food Science and Technology Program, Federal University of Paraíba, João Pessoa, Brazil; 2 Department of Rural Technology, Federal Rural University of Pernambuco, Recife, Brazil; 3 Embrapa - Goats and Sheep, Sobral, Brazil; 4 Bromatology Laboratory, Department of Nutrition, Federal University of Paraíba, João Pessoa, Brazil; 5 Centre for Interdisciplinary Research in Animal Health (CIISA), Faculty of Veterinary Medicine, University of Lisbon, Lisbon, Portugal; 6 Department of Nutrition, Federal University of Campina Grande, Cuité, Brazil; Higher Institute of Applied Sciences and Technology of Gabes University of Gabes, TUNISIA

## Abstract

Goat fat is one of the best sources of conjugated linoleic acid (CLA), a fatty acid which has health benefits. However, though CLA is generated in ruminants, CLA consumption is limited to meats and milk products. This study aimed to replace vegetable fat with goat milk fat enriched with CLA. From differing fat sources, four cookie recipes were developed: CVF–vegetable fat cookies; CB–butter cookies; CG–goat milk fat cookies without CLA; CGCLA–goat milk fat cookies with CLA. The cookies were evaluated using physical (color and texture), physical-chemical parameters (lipids, proteins, total sugars, fiber, ash, moisture and Aw), consumer testing (n = 123), and lipid profiles. The CGCLA presented higher values in the color parameters. The highest and the lowest scores obtained for hardness were respectively 5.54 (CB) and 2.21 (CVF). Lipids and total sugars varied inversely; the highest percentages of lipids were in the CVF and CG samples which obtained lower total sugar content. There were no differences in acceptance or preference for the four formulations. The goat cream formulations (CG and CGCLA) were as well accepted as the CFV formulation. For lipid profiles, CFV presented the highest percentage of trans-fatty acids (TFA) at 16.76%. CGCLA presented 70% more CLA than either CB or CG, certifying that CGCLA presents CLA in relevant quantities, even after cooking. The CGCLA presented higher levels of CLA, and in this study it was verified that goat milk cream enriched with CLA can be used in producing cookies, adding functional and nutritional properties, and offering another alternative(s) to produce food from goat milk fat.

## Introduction

The industrialized trans-fatty acids (iTFA) present in hydrogenated vegetable fat are associated with adverse health impacts, they affect the lipoprotein profile in blood plasma, and are directly related to cardiovascular disease and other chronic non-communicable diseases (NCD). Ruminant trans-fatty acids (TFAr) are TFAs formed through bio-hydrogenation of linoleic and linolenic acids in ruminants or by the enzymatic action of Δ 9-desaturase on vaccenic acid yielding conjugated linoleic acid (CLA) [1]. CLA is scientifically known for having functional anti-carcinogenic, anti-obesity, abdominal fat reducing, anti-atherogenic and immunomodulatory properties [2, 3].

Due to TFAr production in ruminants, CLA is found in the lipids fraction of their milk and meat. Goat milk fat is one of the main sources of CLA, which presents physiological benefits such as lowering: cholesterol (without altering triglycerides), high-density lipoprotein (HDL), alanine aminotransferase (ALT), and aspartate aminotransferase (AST) levels. CLA also induces neurodevelopment, and reduces anxiety and intestinal inflammation in rats [4–6]. Hydrogenated vegetable fat is used industrially in most bakery and confectionery products such as biscuits and cookies. Use of this fat is associated with physicochemical and technological properties such as texture, plasticity, and has a high melting point [7, 8]. Due to the characteristics of iTFA, products such as biscuits and cookies (especially rich in fats) are a challenge to develop if using other lipid sources.

Cookies are a type of biscuit, elaborated in different flavors, which present high levels of sugar and fat; with little water (1–5%) [9, 10]. In this context, the objective of this work was to develop cookies (a type of biscuit) elaborated from goat milk fat enriched with CLA (*cis*9-*trans*11); aiming to obtain an alternative matrix for TFAr consumption, since TFAr is only found in dairy and meat products.

## Material and methods

### Material

The goat milk fats used in the present study were provided by the Brazilian Agricultural Research Company—*EMBRAPA*. Goat milk fat with normal CLA levels and goat milk fat with high CLA levels were used. The goats were fed (both for 20 days) with either a standard diet to obtain fat with a normal CLA content, or a standard diet plus 3.5% soybean oil to produce a natural CLA enriched milk. Subsequently, the milks were collected and skimmed separately to collect the fats which were frozen and stored until elaborations of the cookies [11]. The other ingredients used in the cookie formulations were purchased from Joao Pessoa-PB and Recife-PE markets. Declare that the present study was carried out in accordance by the Research Ethics Committee of the Health Sciences Center of the Federal University of Paraiba—CEP/CSS. The approval number is 459124715.5.0000.5188, and the form of consent was obtain written.

### Lipid profile of fats

Samples of the fats, VF: hydrogenated vegetable fat, B: butter, G: goat milk fat, and GCLA: goat milk fat enriched with CLA were analyzed for their lipid profiles. The trans-esterification procedure for the samples was performed in accordance with Sukhija and Palmquist [12] and Palmquist and Jenkins [13], with adaptations. The fatty acids were analyzed for methyl esters by gas chromatography with flame ionization detection (GC-FID), using a QP2010-plus Shimadzu chromatograph (Shimadzu, Kyoto, Japan) equipped with a silica-fused (SP-2560, 100 m × 0.25 mm × 0.20 μm, Supelco, Bellefonte, PA, USA).

During the analyses, the injector and detector were respectively maintained at 250 °C and 280 °C. Helium was used as the entrainment gas at a constant flow rate of 1 ml/min and 1 μl of sample was injected. The oven temperature was programmed to start at 50 °C, and then was increased by 50 °C per minute until it reached 150 °C, where it remained for 20 minutes, next the temperature was increased by 1 °C per minute until it reached 190 °C for 1 minute, and then increased by 2 °C per minute until reaching 220 °C, and finally remaining at 220 °C for 30 minutes. The fatty acids identified are expressed as percentages and shown in Table 1.

**Table 1:** Fatty acid profile for the different fat sources expressed in 100 mg/fat.

FATTY ACIDS	VF	B	G	GCLA
**SATURATED**				
C4:0	-	3,32^a^ ± 0,50	2,29^b^± 0,43	2,6^b^ ± 0,30
C6:0	-	2,06± 0,20	2,75± 0,15	2,27± 0,27
C8:0	0,01± 0,00	1,19^b^± 0,70	3,27^a^ ± 0,32	1,84^b^ ± 0,40
C10:0	0,01^d^ ± 0,01	2,58^c^ ± 0,20	11,14^a^ ± 1,50	5,28^b^ ± 0,45
C12:0	0,07^d^ ± 0,01	2,98^b^± 0,35	4,37^a^± 0,55	1,95^c^ ± 0,20
C14:0	0,13^c^ ± 0,01	10,91^a^ ± 1,20	10,15^a^ ± 1,01	6,14^b^ ± 0,76
C15:0	0,02^c^ ± 0,00	1,92^a^ ± 0,15	1,38^ab^ ± 0,19	1,01^b^ ± 0,10
C16:0	12,77^c^ ± 1,10	31,01^a^ ± 3,90	25,55^ab^ ± 2,85	21,01^b^ ± 2,5
C17:0	0,03^c^ ± 0,01	1,43^a^ ± 0,20	1,09^b^ ± 0,10	0,97^b^ ± 0,08
C18:0	11,20^b^ ± 1,10	11,32^b^ ± 1,03	12,13^b^ ± 1,20	21,20^a^ ± 2,50
C20:0	0,39^a^ ± 0,05	0,15^b^ ± 0,01	0,29^a^ ± 0,03	0,37^a^ ± 0,05
C21:0	0,03± 0,02	0,03± 0,01	0,04± 0,02	0,04± 0,02
C22:0	0,44^a^ ± 0,04	0,06^b^ ± 0,02	0,07^b^ ± 0,02	0,11^b^ ± 0,03
C23:0	0,05± 0,03	0,03± 0,02	0,02± 0,01	0,02± 0,01
C24:0	0,16^a^± 0,03	0,05^b^ ± 0,02	0,03^b^ ± 0,01	0,02^c^ ± 0,01
TOTAL SFA	25,32^c^ ± 2,95	69,01^ab^ ± 5,50	75,41^a^ ± 7,00	64,81^ab^ ± 6,05
**MONOUNSATURATED**				
C14:1C9	-	0,94 ^a^± 0,07	0,11^b^ ± 0,06	0,05^b^ ± 0,01
C16:1C7	0,01^c^ ± 0,00	0,23^b^± 0,01	0,27^a^ ± 0,02	0,29^a^ ± 0,03
C16:1C9	0,06^c^ ± 0,02	1,38^a^ ± 0,20	0,41^b^± 0,6	0,31^b^ ± 0,04
C17:1C9	0,01^c^± 0,00	0,26^a^± 0,05	0,16^b^± 0,03	0,10^b^± 0,03
C18:1T6+T8	3,98^a^± 0,40	0,26^a^± 0,6	0,16^a^ ± 0,04	0,52^b^ ± 0,06
C18:1T9	3,04^a^ ± 0,30	0,19^c^ ± 0,03	0,14^c^ ± 0,02	0,46^b^ ± 0,05
C18:1T10	7,90^a^± 0,85	0,26^a^ ± 0,03	0,16^d^± 0,01	0,45^b^ ± 0,05
C18:1T11	4,81^a^ ± 0,55	1,90^b^ ± 0,22	0,65^c^ ± 0,07	4,45^a^ ± 0,4
C18:1T12	3,31^a^ ± 0,35	0,26^c^ ± 0,06	0,17^c^ ± 0,03	0,60^b^ ± 0,08
C18:1C9	30,86^a^ ± 4,10	21,41^b^ ± 2,54	19,21^b^ ± 2,20	22,79^b^ ± 2,22
C18:1T15	1,60^a^ ± 0,17	0,18^c^ ± 0,05	0,10^c^ ± 0,3	0,35^b^ ± 0,06
C18:1C11	2,25^a^± 0,33	0,44^b^± 0,07	0,30^c^± 0,05	0,34^b^± 0,03
C18:1C12	8,90^a^± 0,90	0,13^c^± 0,02	0,09^c^± 0,03	0,24^a^± 0,03
C18:1C13	0,46^a^± 0,02	0,06^b^± 0,02	0,02^c^± 0,01	0,05^b^± 0,02
C18:1T16+C14	0,47^a^± 0,04	0,30^b^± 0,04	0,18^c^± 0,04	0,48^a^± 0,04
C18:1C15	0,44^a^± 0,07	0,10^b^± 0,03	0,03^c^± 0,01	0,09^b^ ± 0,02
C20:1	0,16^a^± 0,01	0,04^b^± 0,01	0,04^b^± 0,02	0,06^b^± 0,02
TOTAL MUFA	67,80^a^± 6,50	28,27^b^± 3,77	22,15^c^± 2,60	31,57^b^± 3,50
**POLYUNSATURATED**				
C18:2N6	6,22^a^± 0,95	1,16^a^± 0,50	1,56^b^± 0,65	1,52^b^± 0,55
C18:3N-6	-	0,02± 0,01	0,02± 0,01	0,01± 0,00
C18:3N3	0,20^bc^± 0,03	0,40^a^± 0,04	0,22^c^± 0,02	0,14^b^± 0,03
**CLAC9T11**	-	**0,87**^**b**^**± 0,03**	**0,34**^**c**^ **± 0,01**	**1,69**^**a**^ **± 0,12**
C20:3N-6	-	0,04^a^ ± 0,02	0,02^b^ ± 0,01	0,01^b^ ± 0,00
C20:4N-6	-	0,08^b^ ± 0,03	0,16 ^a^ ± 0,03	0,13^ab^ ± 0,04
C20:5N-3	-	0,03± 0,02	0,02± 0,01	0,01± 0,01
C22:5N-3	-	0,06± 0,02	0,05± 0,01	0,04± 0,02
C22:6N-3	-	0,01± 0,00	0,02± 0,01	0,03± 0,02
TOTAL PUFA	6,42^a^ ± 0,65	2,66^c^± 0,30	2,43^c^ ± 0,25	3,58^b^± 0,30
**TRANS**	25,11^a^ ± 2,02	3,34^c^ ± 0,03	1,54^d^ ± 0,02	7,31^b^ ± 0,06

Data expressed as mean ±standard deviation, statistical analysis performed ANOVA followed by Tukey’s, with (p <0.05), differing letters for VF: hydrogenated vegetable fat; B: butter, G: goat fat, GCLA: goat fat with CLA.

### Cookie production

Four cookie formulations were developed varying the fat source; being CVF—hydrogenated vegetal fat cookies; CB—butter fat cookies; CG—goat milk fat cookies; CGCLA—goat milk fat cookies enriched with CLA. Before the finalized compositions were determined, preliminary tests were carried out until an ideal formulation was agreed upon. The ingredients and quantities used are described in Table 2. After preparation, the cookies were baked for 15 minutes at 165 °C in a conventional oven. Three repetitions were performed; each repetition contained two hundred and forty cookies, with sixty cookies of each variety of fat.

**Table 2:** Ingredients used in the cookie formulations—Expressed in 100 g as fresh matter.

Ingredients (g)	Cookies			
	CVF	CB	CG	CGCLA
Maize starch	17,84	17,84	17,84	17,84
Rice flour	11,74	11,74	11,74	11,74
Hydrogenated Vegetable fat	18,78	-	-	-
Butter without salt	-	18,78	-	-
Goat fat without enrichment the CLA	-	-	18,78	-
Goat fat enrichment with the CLA	-	-	-	18,78
Brown sugar	18,78	18,78	18,78	18,78
Chestnut	11,74	11,75	11,74	11,74
Raisin	9,4	9,4	9,4	9,4
Egg	9,4	9,4	9,4	9,4
Xanthan gun	0,47	0,47	0,47	0,47
Cinnamon powder	0,24	0,24	0,24	0,24
Clove powder	0,24	0,24	0,24	0,24
Yeast chemical	0,94	0,94	0,94	0,94
Salt	0,47	0,47	0,47	0,47

CVF—hydrogenated vegetable fat; CB—butter; CG—goat milk fat; CGCLA—goat milk fat enrichment with the CLA.

### Physical analyses

#### Instrumental color

Determination of the cookies’ instrumental color was performed in a CR-300 Minolta colorimeter (Minolta Co., Osaka, Japan) in accordance with the CIELAB system [14]. In CIELAB, the colorimetric space defined by L*, a*, and b*; the L* coordinate corresponds to the brightness, ranging from 0 (black) to 100 (white), while a* and b* respectively refer to the green (-)/red (+), and blue (-)/yellow (+) chromaticity coordinates. The measurements were performed with the apparatus previously calibrated in reflectance mode with specular reflection excluded and using reference plates. A colon at the top of the cookie is for the measurement; followed by a dot at the bottom.

#### Instrumental texture

Texture determination was performed in a TA.XT Plus texture analyzer from Extralab Brazil. The data obtained were analyzed using StableMicroSystems software/TE32L/Version 6.1.4.0, England. Each sample was placed horizontally on the platform and cut in half with a blade probe (HDP/3PB), pre-test speeds of 1 mm/s and 3 mm/s, and a post-test speed of 10 mm/s, a sheer force of 5x10^-3^ g, and 5.0 mm of distance was used; the force of rupture or breaking (hardness) was recorded.

### Physicochemical analysis

#### Proteins, lipids, total sugars, fibers, ashes and moisture

Protein, total sugar, ash, and moisture contents were determined in accordance with the Association of Official Analytical Chemist Methods [15]. Lipids were determined in accordance with Folch, Lees, and Stanley [16]. Fibers were determined in accordance with Henneberg and Stohmann [17], and water activity (Aw) was determined at 25 °C using Aqualab equipment.

### Sensory evaluation

The sensorial tests were performed after approval by the Research Ethics Committee of the Health Sciences Center at the Federal University of Paraiba, under reference number 052900/2015. For Sensory Analysis; Acceptance, Intention of Purchase, and Ordering tests were performed in accordance with Dutcosky [18], using 123 untrained judges, being 76.42% women and 23.57% men. The samples were coded with random 3-digit numbers and served in plastic containers in an equivalent manner, in complete and balanced blocks. In addition to the samples, each evaluator received a glass with water to clean the palate during the evaluation. The tests were conducted at the Mauricio de Nassau Faculty and the Federal University of Paraiba, in the Dietetic Technique laboratories at each institution, both being in Joao Pessoa. The judges were in perfect health.

The judges received a sheet by which they evaluated each sample for appearance, color, aroma, flavor, texture, and overall evaluation using a hedonic 9 point scale, where is 9 the maximum “like it very much” and 1 the minimum “dislike it very much”, containing intermediate points between the values. For purchase intent, participants responded to a 5-point scale expressing their willingness to consume, buy, or not to buy the product. Where 5 is the maximum “will buy it” and 1 the minimum “not will buy it”, containing intermediate points between the values.

### Lipid profile of cookies

The samples of the cookies elaborated using the four different lipid sources were analyzed for their lipid profiles. The trans-esterification procedure for the samples was carried out in accordance with Sukhija and Palmquist [12] and Palmquist and Jenkins [13], with adaptations. The fatty acids were analyzed according to methyl esters by gas chromatography with flame ionization detection (GC-FID) using a QP2010-plus Shimadzu chromatograph (Shimadzu, Kyoto, Japan) equipped with a silica-fuse (SP-2560, 100 m × 0.25 mm × 0.20 μm, Supelco, Bellefonte, PA, USA). During the analyses the injector and detector were maintained at 250 °C and 280 °C, respectively. Helium was used as the entrainment gas at a constant flow rate of 1 ml/min, and 1 μl of sample was injected. The oven temperature was programmed to start at 50°C, then increased by 50 °C per minute until it reached 150 °C, remaining for 20 minutes, and next increased by 1 °C per minute until it reached 190 °C and remained for 1 minute, and then increased 2 °C per minute until reaching 220 °C, and finally remaining at 220 °C for 30 minutes. The identified fatty acids were expressed as percentages and shown in Table 1.

### Statistical analysis

Physical and physical-chemical analyses were performed in triplicate, the data were submitted to analysis of variance (ANOVA) followed by Tukey’s test to verify significant difference between the samples, considering p <0.05, and using Sigma Stat 3.1 software [19]. All of the data sets were submitted to Principal Component Analysis (PCA) using a correlation matrix and Statistica 7.0 software (Statsoft Inc., Tulsa, OK, USA).

## Results and discussion

### Physical analyses

#### Color

The color data are shown in Table 3. Values of L* above 50 tend to be lighter in color. Only the CB presented a difference in relation to the other formulations, with the lowest value (55.66), while the other treatments were 58.34 (CGCLA), 57.68 (CG), and 57.66 (CVF). Extreme values of L* may interfere with sensory perception, since they are either too dark or too light. An increase in the parameter a* and a decrease in L* indicate darkening in progress, however this darkening, associated to an increase of a* and a decrease of L* was not observed in the cookies [20].

**Table 3:** Physical parameters of cookies with different lipid sources.

Variable	Cookies			
	CVF	CB	CG	CGCLA
**L**	57,66^a^ ±0,87	55,66^b^ ±0,66	57,68^a^ ±0,99	58,34^a^ ±0,91
a*	14,05 ±0,76	14,44 ±0,54	14,29 ±0,84	14,21 ±0,95
b*	29,47^c^ ±0,91	30,57^ab^ ±0,99	29,76^b^ ±0,95	30,89^a^ ±0,94
**Texture (Kg)**	2,21^c^ ±0,45	5,54^a^ ±0,66	4,41^b^±0,42	4,05^b^ ±0,48

Data expressed as mean ±standard deviation, statistical analysis performed ANOVA followed by Tukey’s, with (p <0.05), differing letters for CVF—hydrogenated vegetable fat cookies; CB—butter cookies; CG—goat milk fat cookies; CGCLA—goat milk fat cookies with CLA.

The parameter a* presented no significant differences (p> 0.05), however it did obtain positive values, which are associated with red pigments. Parameter b* obtained a significant difference between the formulations; and the values were positive, being related to yellow pigments. According to Marques [21], positive values of a* and b* are expected in cakes, since they respectively present the red and yellow pigments due to the caramelization of sugars and the Maillard reaction.

The Maillard reaction is an interaction between sugars and amino acids, and together with sugar caramelization they produce brown pigments through the cooking process which are associated with luminosity, and a more reddish, and yellow staining [22]. Variation in L* and b* did not affect sensorial perception of appearance or color (Table 3), as presented in the sub item “Consumer Test”. According to Sharma and Gurjral [23], browning reactions are influenced by other factors such as moisture, water activity, pH, sugars, type and proportion of starch, cooking time, and temperature.

### Instrumental texture

The cookies’ hardness (evaluated by texturometer) is proportional to the force applied to cause deformation. Hardness varies in accordance with; the composition: quality and quantity of flour, sugars, fats, liquids, and other ingredients; with cooking time, temperature, humidity, and storage conditions. Hardness is one of the factors associated with texture to determine the acceptability of foods, it is desirable that its value is not too high [24].

The CB and CVF presented the highest and lowest respective values. Independent of CLA content, the CG and CGCLA did not differ statistically from each other (p> 0.05). An extreme variance in hardness such as very soft or very hard can interfere in the product’s sensorial quality. Fragile and crunchy foods are known to have irregular and irreproducible relationships for force versus deformation [25]. A study carried out with biscuits modified by using oil rich in omega-3, observed that the formulation presenting intermediate hardness also obtained better indices of sensorial acceptance [26]. According to Bertolin et al. [27], high hardness values ​​in texture negatively influenced the global acceptance of biscuits. In this study, interferences in hardness (Table 3) were not observed in relation to the overall evaluation (p> 0.05).

### Physical-chemical analysis

The physico-chemical data are presented in Table 4; CG and CGCLA present significant reductions in lipid content in relation to CVF and CB (p <0.05). However, when total sugars were evaluated, an opposing result was observed in which CG and CGCLA presented higher values than CVF (p <0.05); CB and CGCLA were equivalent (p> 0.05). In relation to humidity, CG and CGCLA presented significant increases in relation to CVF and CB (p <0.05), water activity (Aw) was higher in CG and lower in CVF, presenting a statistical difference between these formulations (p<0.05). Despite the variation in Aw, the formulations presented a value equal to or less than 0.53 Aw and thus, due to an Aw of less than 0.6 they were considered microbiologically stable. Protein, fiber, and ash did not vary significantly between the formulations.

**Table 4:** Physical-chemical parameters of cookies made using different lipid sources—Expressed in 100 g as dry matter.

Variable	Cookies			
	CVF	CB	CG	CGCLA
**Lipids**	28,00^a^ ± 0,00	25,80^b^ ± 0,66	21,16^c^ ± 0,24	20,86^c^ ± 0,68
**Proteins**	5,43 ± 0,25	5,50 ± 0,32	5,90 ± 0,08	5,79 ± 0,30
**Total Sugars**	51,38^c^ ± 0,32	54,42^b^ ± 0,36	56,38^a^ ± 0,33	55,02^b^ ± 0,12
**Fibers**	0,24 ± 0,01	0,25 ± 0,01	0,26 ± 0,01	0,25 ± 0,00
**Ashes**	1,61 ± 0,24	1,70 ± 0,16	1,63 ± 0,27	1,59 ± 0,28
**Moisture**	4,39^c^ ± 0,29	4,64^c^ ± 0,22	6,52^a^ ± 0,23	5,35^b^ ± 0,07
**Water activity (Aw)**	0,35^b^ ± 0,47	0,43^ab^ ± 0,03	0,53^a^ ± 0,17	0,47^ab^ ± 0,26

Data expressed as mean ±standard deviation, statistical analysis performed ANOVA followed by Tukey’s, with (p <0.05), differing letters for CVF—hydrogenated vegetable fat; CB—butter; CG—goat milk fat; CGCLA—goat milk fat with CLA.

The only alteration in the preparation of the cookies was the type of fat used during processing; the amount of fat was 18.78% for all formulations. The observed changes in the physical-chemical composition of the cookies were associated with the type of fat, since only this component varied in the four formulations, the fats having both different processing and compositions. Cream and butter are fat-in-water emulsions; the lipid content may vary from 12 to 60% for cream, and by 80% for butter, the remaining percentages belong to the other compounds like water, sugars, and proteins. Hydrogenated vegetable fat, elaborated in a hydrogenation process, adding hydrogen to a vegetable fat, is formed of 100% lipids [28].

In the case of oatmeal-rich biscuits, the water content of the oatmeal oil is similar to that of oysters. According to Chung, Cho, and Lim [29], the moisture of the biscuit-cookie should vary between 2 and 8 g/100g. The four cookie formulations used were within this variation. Foods have a sigmoid relationship with water and Aw, since moisture content and Aw have a strong effect on the perception of sharpness, and the mechanical sensations of dry and brittle foods as in biscuits [30, 31].

### Sensory evaluation

Evaluation of the mean scores obtained in the sensory analyses (Table 5) showed that the formulations did not differ significantly for the parameters of appearance, color, aroma, flavor, texture, and for the overall evaluation (p> 0.05). Despite the use of different types of fats with different and peculiar characteristics (such as goat cream for the preparation of cookies), these changes were not noticeably perceptible to the judges. In relation to the fat added to the cookies, the formulations were only qualitatively and not quantitatively altered (Table 1), although there was a significant difference (p <0.05) in the lipid content of these formulations (Table 4).

**Table 5:** Mean values of the sensory acceptance and purchase intention tests of cookies prepared from different fat sources.

Attribute	Cookies			
	CVF	CB	CG	CGCLA
**Appearance**	7,47 ±1,36	7,35 ±1,43	7,46 ±1,45	7,32 ±1,38
**Color**	7,62 ±1,18	7,37 ±1,32	7,41 ±1,40	7,40 ±1,29
**Aroma**	6,83 ±1,86	6,83 ±1,70	7,09 ±1,51	6,72 ±1,90
**Flavor**	6,81 ±2,12	6,80 ±1,83	7,15 ±1,67	6,96 ±1,76
**Texture**	7,00 ±1,98	7,03 ±1,58	7,14 ±1,75	7,03 ±1,56
**Overall assessment**	7,20 ±1,62	7,13 ±1,48	7,53 ±1,32	7,10 ±1,60
**Purchase intention**	3,60^ab^ ±1,30	3,52^b^ ±1,22	3,96^a^ ±1,11	3,67^ab^ ±1,26

Data expressed as mean ± standard deviation, statistical analysis performed ANOVA followed by Tukey’s, with (p <0.05), differing letters for CVF—hydrogenated vegetable fat; CB—butter; CG—goat milk fat; CGCLA—goat milk fat with CLA.

When developing biscuits formulated with different concentrations of oats and palm oil, Bertolin et al. [27] found that higher oat concentrations (51%) and lower palm oil concentrations (8%) negatively interfered in the sensorial acceptance of the biscuit. Biguzzi, Schlich and Lange [32], evaluating sensorial perception in biscuits with reductions in fats (by 15% and 25%) as compared to standard biscuits, have observed that the cookies made with the lowest fat percentage (the 25% reduction) revealed significant sensorial differences in relation to the 15% formulation, and the standard formulation.

The studies cited above have reported that variations in the percentages of fat in biscuits are perceptible to consumers, affecting product acceptance [32, 33]. In our study, it was verified that variation of fat type (VF, B, G, and GCLA) was not (to the judges) perceptible; in cookie formulations, G and GCLA could replace VF, without altering sensory acceptance. In the present study, the biscuits elaborated with goat cream presented high purchase intentions, similar to the cookies containing either vegetable fat or goat cream enriched with CLA (p<0.05). Despite this significant difference, the variation of increase to 0.5 in the scores between cookies indicates that the tasters might well buy one of the formulations, regardless of the fat used in their processing.

According to the preference analysis in our research (data not shown), there were no significant differences between the four formulations (p> 0.05). The CG and CGCLA samples were judged as equal to the CVF samples; in the preparation of both industrial cookies and biscuits, VF is most often used [34]. Thus the flavor typical of products of goat origin [35] did not influence the sensory perception of the cookies when compared to the other formulations.

Thus, it was verified that goat milk fat can be used as a substitute for hydrogenated vegetable fat in biscuits; the iTFA present in the VF lipid composition is associated with many diseases, and frequent consumption is related to both predisposition and development of NCD. The use of goat fat in pastry products such as cookies signals potential use and consumption of this fat in other foods in addition to dairy products, adding nutritional, technological, and functional value to the new product.

### Chromatographic analysis of cookies

The CB, CG, and CGCLA presented higher percentages of SFA in comparison to the CVF (Table 6) (p<0.05). However, when comparing CG with CGCLA, it was observed that CGCLA presented fewer SFAs by 7.89%, this reduction was associated to addition of soybean oil to the goats’ diet, altering the lipid profile of the goat cream [11]. The principal component analysis (PCA) positively correlated MUFA and TFA with CVF, and the cookies presented higher percentages of FAs. CLA was positively correlated with CGCLA; a linear association for CL enriched goat fat with CGCLA was observed.

**Table 6:** Fatty acid profile in cookies expressed in 100 mg/fat.

FATTY ACIDS	CVF	CB	CG	CGCLA
**SATURATED**				
C4:0	0,01^c^ ± 0,00	1,29^b^ ± 0,01	1,61^a^± 0,02	1,86^a^ ± 0,01
C6:0	-	1,31^b^ ± 0,01	1,87^a^ ± 0,02	1,54^ab^ ± 0,02
C8:0	0,01^d^ ± 0,00	0,76^c^ ± 0,01	2,10^a^ ± 0,02	1,35^b^ ± 0,01
C10:0	0,02^d^ ± 0,01	1,70^c^ ± 0,01	6,81^a^ ± 0,04	3,57^b^ ± 0,03
C12:0	0,01^d^ ± 0,01	1,97^b^ ± 0,02	2,65^a^ ± 0,02	1,22^c^ ± 0,01
C14:0	0,19^c^ ± 0,00	6,86^a^ ± 0,03	6,14^a^ ± 0,02	3,50^b^ ± 0,01
C15:0	0,04^c^ ± 0,01	0,99^a^ ± 0,01	0,88^a^ ± 0,01	0,62^b^ ± 0,01
C16:0	14,84^c^± 0,90	26,44^a^ ± 1,5	22,93^b^ ± 1,80	19,89^b^ ± 1,05
C17:0	0,09^b^ ± 0,01	0,77^a^ ± 0,03	0,70^a^ ± 0,04	0,57^a^ ± 0,02
C18:0	10,07^b^ ± 0,09	10,93^b^ ± 1,01	11,19^b^ ± 0,09	14,85^a^ ± 1,21
C20:0	0,33^a^ ± 0,03	0,18^c^ ± 0,02	0,25^b^ ± 0,02	0,25^b^ ± 0,02
C21:0	0,02 ± 0,01	0,01 ± 0,00	0,02 ± 0,01	0,02 ± 0,01
C22:0	0,31^a^ ± 0,02	0,05 ^b^ ± 0,01	0,06 ^b^ ± 0,01	0,08 ^b^ ± 0,01
C23:0	0,04 ± 0,01	0,02 ± 0,01	0,02 ± 0,00	0,02 ± 0,01
C24:0	0,13^b^ ± 0,01	0,03^b^ ± 0,01	0,04^b^ ± 0,01	0,03^b^ ± 0,01
TOTAL SFA	26,11^b^ ± 0,01	53,29^a^ ± 4,87	57,26 ^a^ ± 5,40	49,37^a^ ± 3,80
**MONOUNSATURATED**				
C14:1C9	0,01^c^ ± 0,01	0,53^a^ ± 0,01	0,07^b^ ± 0,01	0,04^bc^ ± 0,01
C16:1C7	-	0,43^b^ ± 0,05	0,54^ab^ ± 0,07	0,74^a^ ± 0,10
C16:1C9	0,25^c^± 0,06	1,02^a^± 0,18	0,45^b^± 0,08	0,47^b^± 0,06
C17:1C9	0,04^c^ ± 0,01	0,14^a^ ± 0,05	0,11^a^ ± 0,03	0,08^b^± 0,03
C18:1T6+T8	2,40^a^± 0,54	0,18^c^± 0,05	0,10^c^± 0,3	0,34^b^± 0,10
C18:1T9	2,23^a^ ± 0,40	0,13^c^ ± 0,04	0,10^c^± 0,03	0,32^b^± 0,10
C18:1T10	5,10^a^± 0,48	0,21^bc^± 0,06	0,11^c^± 0,03	0,33^b^± 0,08
C18:1T11	3,31^a^ ± 0,40	0,79^c^ ± 0,09	0,45^d^ ± 0,05	2,47^b^ ± 0,20
C18:1T12	2,11^a^± 0,22	0,20^c^± 0,05	0,12^c^± 0,03	0,33^b^± 0,06
C18:1C9	33,03^a^± 3,43	25,92^b^± 2,40	26,68^b^± 2,22	29,67^b^± 2,54
C18:1T15	1,30^b^ ± 0,15	0,17^b^ ± 0,03	0,10^c^ ± 0,02	0,16^b^± 0,03
C18:1C11	1,79^a^± 0,20	0,61^b^± 0,10	0,55^b^± 0,09	0,62^b^± 0,09
C18:1C12	5,82^a^± 0,60	0,11^c^± 0,03	0,06^c^ ± 0,02	0,26^b^± 0,07
C18:1C13	0,30^a^± 0,08	0,04^b^± 0,02	0,01^c^± 0,00	0,04^b^± 0,02
C18:1T16+C14	0,30^a^± 0,07	0,18^b^± 0,05	0,11^b^± 0,03	0,26^a^± 0,04
C18:1C15	0,29^a^± 0,06	0,06^b^± 0,02	0,02^c^± 0,01	0,05^bc^± 0,02
C20:1	0,13± 0,05	0,06± 0,03	0,06± 0,04	0,07± 0,03
TOTAL MUFA	58,1^a^± 6,00	30,75^bc^± 2,80	29,62^c^± 2,87	36,20^b^± 3,50
**POLYUNSATURATED**				
C18:2N6	15,20^a^± 1,33	15,17^a^± 1,65	12,18^b^± 1,15	12,93^ab^±1,23
C18:3N-6	-	0,02^b^± 0,01	0,20^a^± 0,04	0,01^b^± 0,01
C18:3N3	0,18^b^± 0,03	0,25^a^± 0,05	0,20^ab^± 0,05	0,17^b^± 0,02
**CLAC9T11**	**-**	**0,25**^**b**^**± 0,01**	**0,21**^**b**^ **± 0,01**	**0,95**^**a**^ **± 0,03**
C20:3N-6	0,01± 0,00	0,04± 0,02	0,02± 0,01	0,02± 0,01
C20:4N-6	0,07^b^± 0,02	0,13^ab^± 0,04	0,21^a^± 0,05	0,16^a^± 0,04
C20:5N-3	-	0,01± 0,01	0,01± 0,00	0,01± 0,00
C22:5N-3	-	0,03± 0,02	0,04± 0,01	0,03± 0,01
C22:6N-3	0,02^b^± 0,01	0,03^b^ ± 0,01	0,04^b^ ± 0,02	0,12^a^ ± 0,04
TOTAL PUFA	15,48± 1,35	15,92± 1,40	13,11± 1,11	14,39± 1,20
**TRANS**	16,76^a^ ± 1,30	1,87^c^ ± 0,14	1,09^c^ ± 0,01	4,21^b^ ± 0,03

Data expressed as mean ± standard deviation, statistical analysis performed ANOVA followed by Tukey’s, with (p <0.05), differing letters for CVF—hydrogenated vegetable fat; CB—butter; CG—goat milk fat; CGCLA—goat milk fat with CLA.

It is common for animal fats such as those used in these cookies to be a source of SFA. Palmitic acid was the predominant FA in CB, CB, and CGCLA, representing about 20% of total FA (p<0.05). Capric acid and caprylic acids are characteristic of goat milk fat, being identified in greater proportions in G and GCLA. The decrease in medium chain fatty acids (MCFA) (10 to 16 carbons) is associated with the manipulation of the goats’ diet, since the addition of fatty acid calcium salts to the goat feed can present a decrease in capric and myristic acid, and an increase in linoleic fatty acids (C18: 2 n6), CLA (C18: 2 c9t11), omega-6 (n-6) and PUFAs [36]. In the cookies elaborated in the present research, the decrease of C10: 0, and the increase of CLA were observed in both CG and CGCLA. The fat naturally enriched with CLA used in the elaboration of the CGCLA received similar treatment, the goat ration was augmented with soybean oil [11].

High consumption of SFAs is commonly associated with cardiovascular disease risk factors. However, they should not be excluded from diets since they are necessary for energy synthesis and make up the cell membranes [37]. Short chain saturated fatty acids (4 to 8 carbons) also present beneficial effects for intestinal health due to their rapid uptake and oxidation by colon cells; stimulating cell proliferation in this tissue, and also playing a role in prevention of cardiovascular, cancer, and inflammatory disease [38].

MUFA presented a higher percentage in CGV with 58.11% (p<0.05); these values are related to the TFA, which presented high percentages when compared to CB, CG and CGCLA. The iTFAs present in vegetable fat are associated with NCDs, since the organism lacks the capacity to metabolize these compounds, leading to atheromas. The TFA content ranged from 16.76% (CVF) to 1.1% (CG), with CGCLA being the second highest percentage at 4.21% (p<0.05). CGCLA showed an increase in TFA when compared to CB and CG (p<0.05), this change in lipid profile in the cookies was related to the fat used in their formulation, which presented a higher percentage of essential fatty acids such as CLA.

The high percentage of TFA in the CVF is related to the lipid profile of the VF used in these cookies. The process of vegetable fat hydrogenation increases the percentage of iTFAs, with these fatty acids being found in industrialized foods such as biscuits and wafers [34]. When evaluating the percentages of TFA in these processed foods in Portugal, Costa et al. [7] observed that Trans-C18:1 and its isomers were present in higher amounts. Elaidic acid (C18:1 trans-9) is the main constituent of iTFA. Oleic acid (C18:1) is a MUFA present in vegetable oils. After hydrogenation, oleic acid changes its spatial and biochemical conformation; forming elaidic acid [39]. Such iTFAs are associated with cardiovascular diseases, obesity, and NCD [40].

The percentages of PUFA presented little quantitative variation, with the highest percentage being CVF (15.48%); the lowest was CG (13.10%). However, the lipid profile differed qualitatively between the formulations. For PUFAs, CGCLA and CG presented higher percentages of docosahexaenoic acid (DHA) (C22:6n-3), which is associated with cardio-protective, neurological development, and anti-inflammatory properties [26].

Another essential PUFA is CLA (C18:2 c9t11), which is only present in ruminant fats, since isomerization and bio-hydrogenation of linoleic acid (C18:2) and linolenic acid (C18:3) only occur in the rumen or mammary glands of these animals. Meat and dairy products are the main sources of TFAr [41, 42]. This natural trans-fat derived from ruminants exerts functional health properties such as plasma antioxidant activity, antimutagenic and anticarcinogenic action, and promoting cytotoxic agent reductions in cancer cells. CLA also triggers immune response stimuli against atherosclerosis, diabetes mellitus, and obesity [3, 43].

After preparation of the cookies, there was a CLA content reduction of 74% compared to CLA enriched fat before cooking. This loss occurred in all of the cookies containing CLA. However, the CGCLA still maintained a higher CLA content; by 70% as compared to the others. Therefore, cookies with CLA enriched goat fat are an alternative for consumption of this essential fatty acid, being that CLA is usually consumed through dairy and meat products. Cookies with CLA help to diversify and add value to products of goat origin; and can help to produce a food with functional potential.

### Principal component analysis

**Fig 1:**
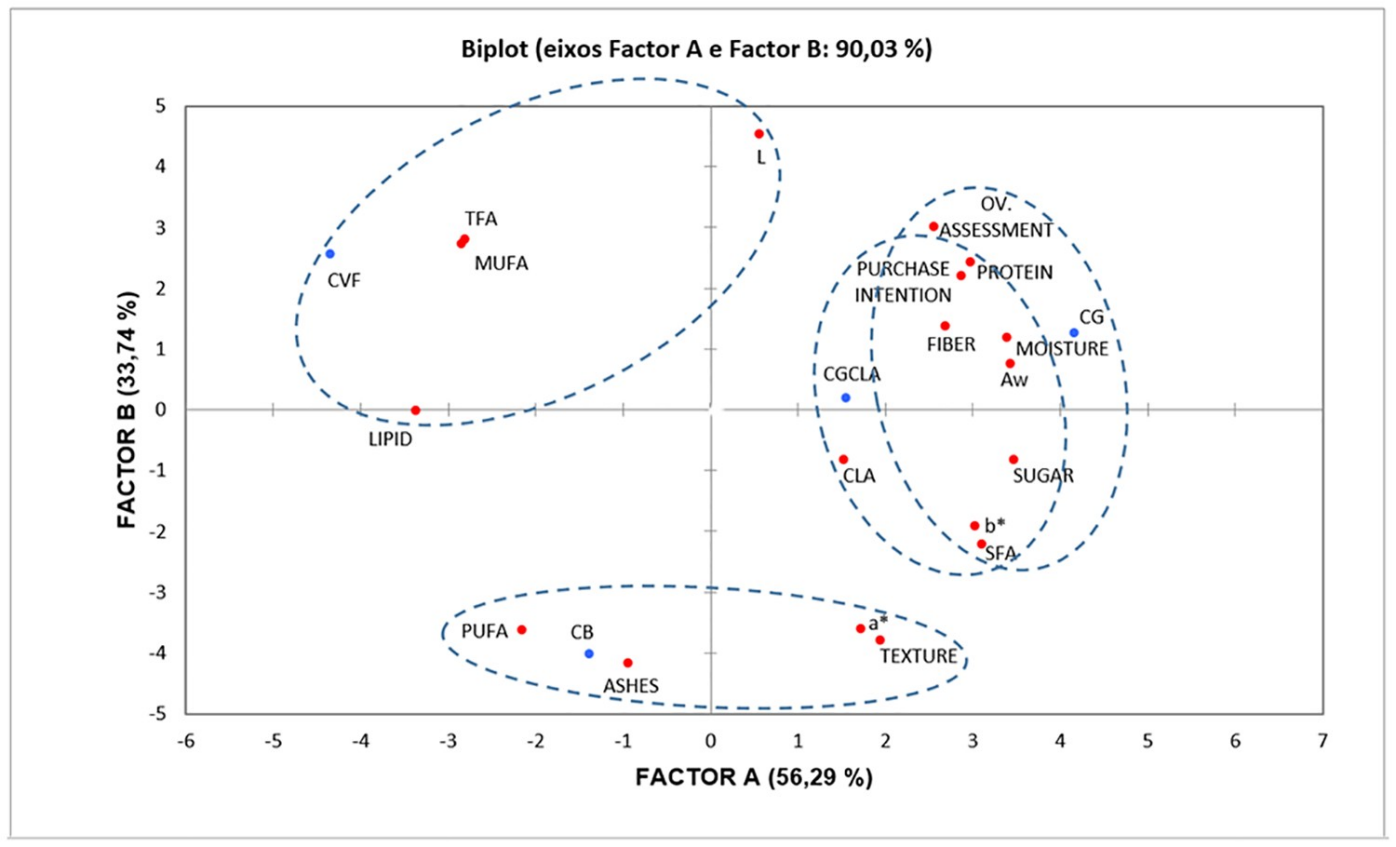
PCA for the physical-chemical, physical, sensory, and chromatographic data of the cookies. CVF (vegetable fat cookie); CB (butter cookie); CG (goat fat cookie) and CGCLA (goat fat-CLA cookie).

Principal Component Analysis (PCA), a multivariate statistical analysis, uses vectors to correlate variables from a set of linear variables, called principal components, aiding in the grouping of data [44]. Fig 1 shows the resulting PCA graph where the first two factors explain 90.03% of the variance, allowing relevant discrimination of the samples in function of their evaluated attributes [45, 46]. The first factor (A) explained 56.2% of the variance, being positively characterized by "b*", "proteins", "sugars", "moisture", "ashes", "fibers", "Aw", “purchases", "SFA", “overall assessment" and "purchase intention", and negatively by "lipids", "MUFA" and “TFA”. The second factor (B) explained 33.74% of the total variance, relating positively with "L", and negatively with "texture", "ashes", and "PUFA".

The analysis was able to separate the samples according to similar characteristics, through the correlations between variables and factors, as well the correlations between sample and factors. It was possible to construct the clusters, as indicated on the PCA graph, showing the presence of three distinct groups. The first composed by the CFV, the second composed by the CG and CGCLA. This last group presented as differences only the attributes CLA and overall assessment, which had a higher correlation with the CGCLA and CG, respectively. CVF formulation correlated with monounsaturated and trans-fatty acids. Whereas CVF was made with hydrogenated vegetable fat rich in monounsaturated and trans-fatty acids. CB was correlated with polyunsaturated fatty acids and ashes. The CG and CGCLA formulations obtained more attributes in common such as Aw, moisture, proteins, fibers, purchase intention, and overall evaluation. These similar characteristics can be associated with goat cream, the same fat used to make CG and CGCLA, varying only the CLA content, between G and GCLA. CGCLA correlated with CLA, the formulation presented the highest percentage of this PUFA.

## Conclusion

Substitution of hydrogenated vegetable fat and butter with goat cream in cookies is feasible, since it does not alter the flavor (sensorial acceptance), improves the fatty acid profile, and increases the levels of essential fatty acids like CLA. This improves its biological properties, and may increase demand by consumers seeking to eat tasty foods that include functional potential. Considering the metabolic and behavioral benefits of CLA, such cookies are vehicles for its consumption; promoting health benefits. However, *in vivo* studies are needed to evaluate dosages, daily consumption, systemic effect, and health benefits.

## Supporting information

S1 Table Fatty acid profile for the different fat sources; expressed in 100g/ fat. Data expressed as mean ±standard deviation, statistical analysis performed ANOVA followed by Tukey’s, with (p <0.05), differing letters for VF: hydrogenated vegetable fat; B: butter, G: goat fat, GCLA: goat fat with CLA.
(DOCX)

S2 Table Ingredients used in the cookie formulations—Expressed in 100 g. CVF—hydrogenated vegetable fat; CB—butter; CG—goat milk fat; CGCLA—goat milk fat enrichment with the CLA.
(DOCX)

S3 Table Physical parameters of cookies with different lipid sources. Data expressed as mean ±standard deviation, statistical analysis performed ANOVA followed by Tukey’s, with (p <0.05), differing letters for CVF—hydrogenated vegetable fat cookies; CB—butter cookies; CG—goat milk fat cookies; CGCLA—goat milk fat cookies with CLA.
(DOCX)

S4 Table Physical-chemical parameters of cookies made using different lipid sources. Data expressed as mean ±standard deviation, statistical analysis performed ANOVA followed by Tukey’s, with (p <0.05), differing letters for CVF—hydrogenated vegetable fat; CB—butter; CG—goat milk fat; CGCLA—goat milk fat with CLA.
(DOCX)

S5 Table Mean values of the sensory acceptance and purchase intention tests of cookies prepared from different fat sources. Data expressed as mean ± standard deviation, statistical analysis performed ANOVA followed by Tukey’s, with (p <0.05), differing letters for CVF—hydrogenated vegetable fat; CB—butter; CG—goat milk fat; CGCLA—goat milk fat with CLA.
(DOCX)

S6 Table Fatty acid profile in cookies expressed in 100 g/fat. Data expressed as mean ± standard deviation, statistical analysis performed ANOVA followed by Tukey, with different letters for (p <0.05). CVF—hydrogenated vegetable fat; CB—butter; CG—goat’s milk fat; CGCLA—goat’s milk fat with CLA.
(DOCX)

S1 Fig PCA for the physical-chemical, physical, sensory, and chromatographic data of the cookies. CVF (cookie with hydrogenated vegetable fat), CB (cookie with butter), CG (cookie with goat cream) and CGCLA (cookie with enriched goat cream with CLA).
(DOCX)

## References

[1] Mohamed H, El-Salam ABD, El-Shibiny S. Conjugated linoleic acid and vaccenic acid contents in cheeses: An overview from the literature. J. Food Compos. Anal. 2014; 33: 117–126.

[2] Koba K, Yanagita T. Health benefits of conjugated linoleic acid (CLA). Obes Res Clin Pract. 2014; 8:525–532. 10.1016/j.orcp.2013.10.001 25434907

[3] Park Y, Pariza M, The bioactivities and potential mechanisms of action of the conjugated linoleic acid. Food Sci Biotechnol. 2009; 18: 586–593.

[4] Lopez-Aliaga I, Alferez MJM, Nestares MT, Ros PB, Barrionuevo M, Campos MS. Goat milk feeding causes an increase in biliary secretion of cholesterol and a decrease in plasma cholesterol levels in rats. J. Dairy Sci. 2005; 88: 1024–1030. 10.3168/jds.S0022-0302(05)72770-3 15738237

[5] Soares JKB, Melo APR, Medeiros MAC, Queiroga RCRE, Bomfim MAD, Santiago ECA et al Anxiety behavior is reduced, and physical growth is improved in theprogeny of rat dams that consumed lipids from goat milk: An elevatedplus maze analysis. Neurosci. Lett. 2013;. 552: 25–29. 10.1016/j.neulet.2013.07.028 23916660

[6] Assis POA, Guerra JCB, Araujo DFS, Junior RFA, Machado TADG, Araujo AA, et al Intestinal anti-inflammatory activity of goat milk and goat yoghurt in the acetic acid model of rat colitis. Int Dairy J. 2016; 56: 45–54.

[7] Costa N, Cruz R, Graça P, Breda J, Casal S. Trans fatty acids in the Portuguese food market. Food Control. 2016; 64: 128–134. 10.1016/j.foodcont.2015.12.010 27274619 PMC4763144

[8] Tarancón P, Fiszman SM, Salvador A, Tárrega A. Formulating biscuit with healthier fats. Consumer profiling of textural and flavor sensations during consumption. Food Res. Int. 2013; 53 (1): 134–140.

[9] Pareyt B, Talhaoui F, Kerckhofs G, Brijs K, Goesaert H, et al The role of sugar and fat in sugar-snap cookies: Structural and textural properties. J Food Eng. 2009; 90 (3) 400–408.

[10] Gökmen V, Serpen A, Açar OÇ, Morales FJ. Significance of furosine as eat-induced marker in cookies. J Cereal Sci. 2008; 48 (3): 843–847.

[11] Barbosa MQ, Queiroga RCRE, Bertozzo CCMS, Araújo DFS, Oliveira LIG, Silva JYP et al Effect of diets with goat milk fat supplemented with exercise on anxiety and oxidative stress in the brains of adult rats. Food Functional. 2018; 9: 2891–2901. 10.1039/c7fo01764b 29717304

[12] Sukhija PS, Palmquist DL. Rapid method for determination of total fatty acid content and composition of feedstuffs and feces. J Agric Food Chem. 1988; v: 36 -1202–1206.

[13] Palmquist DL, Jenkins TC. Challenges with fats and fatty acid methods. J Anim Sci. 2003; 12: 3250–3254. 10.2527/2003.81123250x 14677882

[14] Commission International de L’eclairage (CIE): “Recommendations on Uniform Color Spaces, Color Difference Equations, Psychometric Color Terms”. Supplement No. 2 to CIE Publication No. 15, Colorimetry, Bureau Central de la CIE, Paris, 1978.

[15] Association of Official Agricultural Chemists. Official methods of analysis of Association of Official Agricultural Chemists. 19^th^ ed, Ass. Off. Analytical. Chem, Washington, USA 2012.

[16] Folch J, Lees M, Stanley GHS. A simple method for the isolation and purification of total lipids from animal tissues. J Biol Chem. 1957; 226: 497–509. 13428781

[17] Henneberg W, Stohmann F. Beiträge zur begründung einer rationallen Fütterung der Wiederkäuer. Praktisch-landwirthschaftliche und chemisch-physiologische Untersuchungen, für Landwirthe und Physiologen. Ueber die Ausnutzung der Futterstoffe durch das volljährige Rind und über Fleischbildung im Körper desselben C. A. Schwetschke und Sohn, Braunschweig, Berlin, Germany, 1864.

[18] Dutcosky SD. Análise sensorial de alimentos. Curitiba: Champagnat, 4 ed; 2013.

[19] Sigmastat (programa de computador). Versão 3.1. 2009; Point Richmond (Califórnia): Comercial.

[20] Lara E, Cortes P, Briones V, Perez M. Structural and physical modifications of corn biscuits during baking process. LWT. 2011; 44: 622–630.

[21] Marques GA, Brilhante JFB, Silva DA, Silva EMN. Whey protein as a substitute for wheat in the development of no added sugar cookies. LWT. 2016; 67: 118–126.

[22] Laguna L, Salvador A, Sanz T, Fiszman SM. Performance of a resistant starch rich ingredient in the baking and eating quality of short-dough biscuits. LWT. 2011; 44: 737–746.

[23] Sharma P, Gujral HS. Extrusion of hulled barley affecting b-glucan and properties of extrudates. Food Bioprocess Tech. 2013; 6: 1374–1389.

[24] Bick MA, Fogaça AO. Cookies with different concentrations of quinoa flour in partial replacement of wheat flour. Braz. J. Food Technol. v. 17, n. 2, p. 121–129, 2014.

[25] Peleg M, Normand MD. Symmetrized dot-patterns (SDP) of irregular compressive stress strain relationship. J. Texture Stud. 1992; 23: 427–438.

[26] Umesha SS, Sai MR, Indiramma AR, Akshitha S, Naidu KA. Enrichment of biscuits with microencapsulated omega-3 fatty acid (Alpha-linolenic acid) rich Garden cress (*Lepidium sativum*) seed oil: Physical, sensory and storage quality characteristics of biscuits. LWT. 2015; 62: 654–661.

[27] Bertolin TE, Centenaro A, Giacomelli B, Reinehr C, Gutkoski LC. Elaboration of biscuits with oatmeal and fat palm with added L-leucine and calcium for sarcopenia. LWT. 2013; 33: (2) 345–354.

[28] Ripolles D, Parron JA, Calvo M, Perez MD, Fitzgerald RJ, Sanchez L. Antioxidant activity of co-products from milk fat processing and their enzymatic hydrolysates obtained with different proteolytic preparations. Int Dairy J. 2016; 60: 70–77.

[29] Chung H, Cho A, Lim S. Utilization of germinated and heat-moisture treated brown rices in sugar-snap cookies. LWT. 2014; 57: 260–266.

[30] Arimi JM, Duggan E, O’sullivan M, Lyng JG, O’riordan ED. Effect of water activity on the crispiness of a biscuit (Crackerbread): Mechanical and acoustic evaluation. Food Res. Int. 2010; 43:1650–1655.

[31] Laguna L, Primo-martín C, Varela P, Salvador A, Sanz T. HPMC and inulin as fat replacers in biscuits: Sensory and instrumental evaluation. LWT. 2014; 56: 494–501.

[32] Biguzzi C, Schlich P, Lange C. The impact of sugar and fat reduction on perception and liking of biscuits. Food Qual Prefer. 2014; 35: 41–47.

[33] Drewnowski A, Nordensten K, Dwyer J. Replacing sugar and fat in cookies: Impact on product quality and preference. Food Qual Prefer. 1998; 9: 13–20.

[34] Santos LAT, Cruz R, Casal S. Trans fatty acids in commercial cookies and biscuits: An update of Portuguese Market. Food Control. 2015; 47:141–146.

[35] Verruck S, Dantas A, Prudencio ES. Functionality of the components from goat’s milk, recent advances for functional dairy products development and its implications on human health. J. of Funct. Food. v. 52, 243–257, 2019.

[36] Gomes LC, Alcalde CR, Santos GT, Feihrmann AC, Molina BSL, Grande PA et al Concentrate with calcium salts of fatty acids increases the concentration of polyunsaturated fatty acids in milk produced by dairy goats. Small Rumin. Res. 2015; 124: 81–88.

[37] Ruiz-Nunez BDA, Dijck-Brouwer J, Muskiet FAJ. The relation of saturated fatty acids with low-grade inflammation and cardiovascular disease. J. Nutri. Biochem. 2016; 36: 1–20. 10.1016/j.jnutbio.2015.12.007 27692243

[38] Gómez-Cortés P, Juárez M, Fuente MA. Milk fatty acids and potential health benefits: An updated vision. Trends Food Sci Technol. 2018; 81: 1–9.

[39] Ganguly R, Pierce GN. The toxicity of dietary trans fats. Food Chem Toxicol. 2015; 78: 170–176. 10.1016/j.fct.2015.02.004 25684416

[40] Ganguly R, Lavallee R, Maddaford TG, Devaney B, Bassett CMC, et al Ruminant and industrial trans-fatty acid uptake in the heart. J. Nutriti. Biochem.2016; 31: 60–66. 10.1016/j.jnutbio.2015.12.018 27133424

[41] Park Y. Conjugated linoleic acid (CLA): Good or bad trans fat?. J. Food Composit Anal. 2009; 22: 4–12.

[42] Mele MC, Cannelli G, Carta G, Cordeddu L, Melis MP, Murro E et al Metabolism of c9,t11-conjugated linoleic acid (CLA) in humans. Prostaglandins Leukot. Essent. Fatty Acids. 2013; 89: 115–119. 10.1016/j.plefa.2013.05.005 23809328

[43] Razzaghia A, Naseriana AA, Valizadeha R, Ebrahimia SH.; Khorramia B, Malekkhahia M et al Pomegranate seed pulp, pistachio hulls, and tomato pomace as replacement of wheat bran increased milk conjugated linoleic acid concentrations without adverse effects on ruminal fermentation and performance of Saanen dairy goats. Anim Feed Sci Technol. 2015; 210: 46–55.

[44] Tran NM, Burdejová P, Ospienko M, Härdle WK. Principal component analysis in an asymmetric norm. Journal Multivariate Anal. 2019; 171: 1–21.

[45] Piclin N, Pintore M, Lanza CM, Scacco A, Guccione S, Giurato L et al Sensory analysis of red wines: discrimination by adaptive fuzzy partition. J Sens Stud. 2008; 23: 58–569.

[46] Nurgel C, Pickering GJ, Inglis DL. Sensory and chemical characteristics of Canadian ice wines. J Sci Agric. 2004; 84: 1675–1684.

