# Supplementary material for: Developing cookies formulated with goat cream enriched with conjugated linoleic acid
Source: PLoS One. 2019 Sep 23;14(9):e0212534. doi: 10.1371/journal.pone.0212534 (PMC6756519; doi:10.1371/journal.pone.0212534)
Supplement: S1 Table — Data expressed as mean ±standard deviation, statistical analysis performed ANOVA followed by Tukey’s, with (p <0.05), differing letters for VF: hydrogenated vegetable fat; B: butter, G: goat fat, GCLA: goat fat with CLA. (DOCX) [file pone.0212534.s001.docx]

**Table 1**. **Fatty acid profile for the different fat sources expressed in 100 mg/fat.**

| FATTY ACIDS | VF | B | G | GCLA |
| --- | --- | --- | --- | --- |
| SATURATED |  |  |  |  |
| C4:0 | - | 3,32^a^ ± 0,50 | 2,29^b^± 0,43 | 2,6^b^ ± 0,30 |
| C6:0 | - | 2,06± 0,20 | 2,75± 0,15 | 2,27± 0,27 |
| C8:0 | 0,01± 0,00 | 1,19^b^± 0,70 | 3,27^a^ ± 0,32 | 1,84^b^ ± 0,40 |
| C10:0 | 0,01^d^ ± 0,01 | 2,58^c^ ± 0,20 | 11,14^a^ ± 1,50 | 5,28^b^ ± 0,45 |
| C12:0 | 0,07^d^ ± 0,01 | 2,98^b^± 0,35 | 4,37^a^± 0,55 | 1,95^c^ ± 0,20 |
| C14:0 | 0,13^c^ ± 0,01 | 10,91^a^ ± 1,20 | 10,15^a^ ± 1,01 | 6,14^b^ ± 0,76 |
| C15:0 | 0,02^c^ ± 0,00 | 1,92^a^ ± 0,15 | 1,38^ab^ ± 0,19 | 1,01^b^ ± 0,10 |
| C16:0 | 12,77^c^ ± 1,10 | 31,01^a^ ± 3,90 | 25,55^ab^ ± 2,85 | 21,01^b^ ± 2,5 |
| C17:0 | 0,03^c^ ± 0,01 | 1,43^a^ ± 0,20 | 1,09^b^ ± 0,10 | 0,97^b^ ± 0,08 |
| C18:0 | 11,20^b^ ± 1,10 | 11,32^b^ ± 1,03 | 12,13^b^ ± 1,20 | 21,20^a^ ± 2,50 |
| C20:0 | 0,39^a^ ± 0,05 | 0,15^b^ ± 0,01 | 0,29^a^ ± 0,03 | 0,37^a^ ± 0,05 |
| C21:0 | 0,03± 0,02 | 0,03± 0,01 | 0,04± 0,02 | 0,04± 0,02 |
| C22:0 | 0,44^a^ ± 0,04 | 0,06^b^ ± 0,02 | 0,07^b^ ± 0,02 | 0,11^b^ ± 0,03 |
| C23:0 | 0,05± 0,03 | 0,03± 0,02 | 0,02± 0,01 | 0,02± 0,01 |
| C24:0 | 0,16^a^± 0,03 | 0,05^b^ ± 0,02 | 0,03^b^ ± 0,01 | 0,02^c^ ± 0,01 |
| TOTAL SFA | 25,32^c^ ± 2,95 | 69,01^ab^ ± 5,50 | 75,41^a^ ± 7,00 | 64,81^ab^ ± 6,05 |
| MONOUNSATURATED |  |  |  |  |
| C14:1c9 | - | 0,94 ^a^± 0,07 | 0,11^b^ ± 0,06 | 0,05^b^ ± 0,01 |
| C16:1c7 | 0,01^c^ ± 0,00 | 0,23^b^± 0,01 | 0,27^a^ ± 0,02 | 0,29^a^ ± 0,03 |
| C16:1c9 | 0,06^c^ ± 0,02 | 1,38^a^ ± 0,20 | 0,41^b^± 0,6 | 0,31^b^ ± 0,04 |
| C17:1c9 | 0,01^c^± 0,00 | 0,26^a^± 0,05 | 0,16^b^± 0,03 | 0,10^b^± 0,03 |
| C18:1t6+t8 | 3,98^a^± 0,40 | 0,26^a^± 0,6 | 0,16^a^ ± 0,04 | 0,52^b^ ± 0,06 |
| C18:1t9 | 3,04^a^ ± 0,30 | 0,19^c^ ± 0,03 | 0,14^c^ ± 0,02 | 0,46^b^ ± 0,05 |
| C18:1t10 | 7,90^a^± 0,85 | 0,26^a^ ± 0,03 | 0,16^d^± 0,01 | 0,45^b^ ± 0,05 |
| C18:1t11 | 4,81^a^ ± 0,55 | 1,90^b^ ± 0,22 | 0,65^c^ ± 0,07 | 4,45^a^ ± 0,4 |
| C18:1t12 | 3,31^a^ ± 0,35 | 0,26^c^ ± 0,06 | 0,17^c^ ± 0,03 | 0,60^b^ ± 0,08 |
| C18:1c9 | 30,86^a^ ± 4,10 | 21,41^b^ ± 2,54 | 19,21^b^ ± 2,20 | 22,79^b^ ± 2,22 |
| C18:1t15 | 1,60^a^ ± 0,17 | 0,18^c^ ± 0,05 | 0,10^c^ ± 0,3 | 0,35^b^ ± 0,06 |
| C18:1c11 | 2,25^a^± 0,33 | 0,44^b^± 0,07 | 0,30^c^± 0,05 | 0,34^b^± 0,03 |
| C18:1c12 | 8,90^a^± 0,90 | 0,13^c^± 0,02 | 0,09^c^± 0,03 | 0,24^a^± 0,03 |
| C18:1c13 | 0,46^a^± 0,02 | 0,06^b^± 0,02 | 0,02^c^± 0,01 | 0,05^b^± 0,02 |
| C18:1t16+c14 | 0,47^a^± 0,04 | 0,30^b^± 0,04 | 0,18^c^± 0,04 | 0,48^a^± 0,04 |
| C18:1c15 | 0,44^a^± 0,07 | 0,10^b^± 0,03 | 0,03^c^± 0,01 | 0,09^b^ ± 0,02 |
| C20:1 | 0,16^a^± 0,01 | 0,04^b^± 0,01 | 0,04^b^± 0,02 | 0,06^b^± 0,02 |
| TOTAL MUFA | 67,80^a^± 6,50 | 28,27^b^± 3,77 | 22,15^c^± 2,60 | 31,57^b^± 3,50 |
| POLYUNSATURATED |  |  |  |  |
| C18:2n6 | 6,22^a^± 0,95 | 1,16^a^± 0,50 | 1,56^b^± 0,65 | 1,52^b^± 0,55 |
| C18:3n-6 | - | 0,02± 0,01 | 0,02± 0,01 | 0,01± 0,00 |
| C18:3n3 | 0,20^bc^± 0,03 | 0,40^a^± 0,04 | 0,22^c^± 0,02 | 0,14^b^± 0,03 |
| CLAc9t11 | - | **0,87^b^± 0,03** | **0,34^c^ ± 0,01** | **1,69^a^ ± 0,12** |
| C20:3n-6 | - | 0,04^a^ ± 0,02 | 0,02^b^ ± 0,01 | 0,01^b^ ± 0,00 |
| C20:4n-6 | - | 0,08^b^ ± 0,03 | 0,16 ^a^ ± 0,03 | 0,13^ab^ ± 0,04 |
| C20:5n-3 | - | 0,03± 0,02 | 0,02± 0,01 | 0,01± 0,01 |
| C22:5n-3 | - | 0,06± 0,02 | 0,05± 0,01 | 0,04± 0,02 |
| C22:6n-3 | - | 0,01± 0,00 | 0,02± 0,01 | 0,03± 0,02 |
| TOTAL PUFA | 6,42^a^ ± 0,65 | 2,66^c^± 0,30 | 2,43^c^ ± 0,25 | 3,58^b^± 0,30 |
| TRANS | 25,11^a^ ± 2,02 | 3,34^c^ ± 0,03 | 1,54^d^ ± 0,02 | 7,31^b^ ± 0,06 |
