# Supplementary material for: Developing cookies formulated with goat cream enriched with conjugated linoleic acid
Source: PLoS One. 2019 Sep 23;14(9):e0212534. doi: 10.1371/journal.pone.0212534 (PMC6756519; doi:10.1371/journal.pone.0212534)
Supplement: S2 Table — CVF—hydrogenated vegetable fat; CB—butter; CG—goat milk fat; CGCLA—goat milk fat enrichment with the CLA. (DOCX) [file pone.0212534.s002.docx]

**Table 2.** **Ingredients used in the cookie formulations - expressed in 100 g as fresh matter.**

|  | **Cookies** | | |  |
| --- | --- | --- | --- | --- |
| **Ingredients (g)** | **CVF** | **CB** | **CG** | **CGCLA** |
| Maize starch | 17,84 | 17,84 | 17,84 | 17,84 |
| Rice flour | 11,74 | 11,74 | 11,74 | 11,74 |
| Hydrogenated Vegetable fat | 18,78 | - | - | - |
| Butter without salt | - | 18,78 | - | - |
| Goat fat whitout enrichment the CLA | - | - | 18,78 | - |
| Goat fat enrichment with the CLA | - | - | - | 18,78 |
| Brown sugar | 18,78 | 18,78 | 18,78 | 18,78 |
| Chestnut | 11,74 | 11,75 | 11,74 | 11,74 |
| Raisin | 9,4 | 9,4 | 9,4 | 9,4 |
| Egg | 9,4 | 9,4 | 9,4 | 9,4 |
| Xanthan gun | 0,47 | 0,47 | 0,47 | 0,47 |
| Cinnamon powder | 0,24 | 0,24 | 0,24 | 0,24 |
| Clove powder | 0,24 | 0,24 | 0,24 | 0,24 |
| Yeast chemical | 0,94 | 0,94 | 0,94 | 0,94 |
| Salt | 0,47 | 0,47 | 0,47 | 0,47 |
