# Supplementary material for: Developing cookies formulated with goat cream enriched with conjugated linoleic acid
Source: PLoS One. 2019 Sep 23;14(9):e0212534. doi: 10.1371/journal.pone.0212534 (PMC6756519; doi:10.1371/journal.pone.0212534)
Supplement: S3 Table — Data expressed as mean ±standard deviation, statistical analysis performed ANOVA followed by Tukey’s, with (p <0.05), differing letters for CVF—hydrogenated vegetable fat cookies; CB—butter cookies; CG—goat milk fat cookies; CGCLA—goat milk fat cookies with CLA. (DOCX) [file pone.0212534.s003.docx]

**Table 3. Physical parameters of cookies with different lipid sources.**

| **Variable** | **Cookies** | | |  |
| --- | --- | --- | --- | --- |
|  | **CVF** | **CB** | **CG** | **CGCLA** |
| **L** | 57,66^a^ ±0,87 | 55,66^b^ ±0,66 | 57,68^a^ ±0,99 | 58,34^a^ ±0,91 |
| **a*** | 14,05 ±0,76 | 14,44 ±0,54 | 14,29 ±0,84 | 14,21 ±0,95 |
| **b*** | 29,47^c^ ±0,91 | 30,57^ab^ ±0,99 | 29,76^b^ ±0,95 | 30,89^a^ ±0,94 |
| **Texture (Kg)** | 2,21^c^ ±0,45 | 5,54^a^ ±0,66 | 4,41^b^±0,42 | 4,05^b^ ±0,48 |
