# Supplementary material for: Developing cookies formulated with goat cream enriched with conjugated linoleic acid
Source: PLoS One. 2019 Sep 23;14(9):e0212534. doi: 10.1371/journal.pone.0212534 (PMC6756519; doi:10.1371/journal.pone.0212534)
Supplement: S4 Table — Data expressed as mean ±standard deviation, statistical analysis performed ANOVA followed by Tukey’s, with (p <0.05), differing letters for CVF—hydrogenated vegetable fat; CB—butter; CG—goat milk fat; CGCLA—goat milk fat with CLA. (DOCX) [file pone.0212534.s004.docx]

**Table 4. Physical-chemical parameters of cookies made using different lipid sources - expressed in 100 g as dry matter.**

| **Variable** | **Cookies** | | |  |
| --- | --- | --- | --- | --- |
|  | **CVF** | **CB** | **CG** | **CGCLA** |
| **Lipids** | 28,00^a^ ± 0,00 | 25,80^b^ ± 0,66 | 21,16^c^ ± 0,24 | 20,86^c^ ± 0,68 |
| **Proteins** | 5,43 ± 0,25 | 5,50 ± 0,32 | 5,90 ± 0,08 | 5,79 ± 0,30 |
| **Total Sugars** | 51,38^c^ ± 0,32 | 54,42^b^ ± 0,36 | 56,38^a^ ± 0,33 | 55,02^b^ ± 0,12 |
| **Fibers** | 0,24 ± 0,01 | 0,25 ± 0,01 | 0,26 ± 0,01 | 0,25 ± 0,00 |
| **Ashes** | 1,61 ± 0,24 | 1,70 ± 0,16 | 1,63 ± 0,27 | 1,59 ± 0,28 |
| **Moisture** | 4,39^c^ ± 0,29 | 4,64^c^ ± 0,22 | 6,52^a^ ± 0,23 | 5,35^b^ ± 0,07 |
| **Water activity (Aw)** | 0,35^b^ ± 0,47 | 0,43^ab^ ± 0,03 | 0,53^a^ ± 0,17 | 0,47^ab^ ± 0,26 |
