# Supplementary material for: Developing cookies formulated with goat cream enriched with conjugated linoleic acid
Source: PLoS One. 2019 Sep 23;14(9):e0212534. doi: 10.1371/journal.pone.0212534 (PMC6756519; doi:10.1371/journal.pone.0212534)
Supplement: S5 Table — Data expressed as mean ± standard deviation, statistical analysis performed ANOVA followed by Tukey’s, with (p <0.05), differing letters for CVF—hydrogenated vegetable fat; CB—butter; CG—goat milk fat; CGCLA—goat milk fat with CLA. (DOCX) [file pone.0212534.s005.docx]

**Table 5. Mean values of the sensory acceptance and purchase intention tests of cookies prepared from different fat sources.**

| **Attribute** | **Cookies** | | |  |
| --- | --- | --- | --- | --- |
|  | **CVF** | **CB** | **CG** | **CGCLA** |
| **Appearance** | 7,47 ±1,36 | 7,35 ±1,43 | 7,46 ±1,45 | 7,32 ±1,38 |
| **Color** | 7,62 ±1,18 | 7,37 ±1,32 | 7,41 ±1,40 | 7,40 ±1,29 |
| **Aroma** | 6,83 ±1,86 | 6,83 ±1,70 | 7,09 ±1,51 | 6,72 ±1,90 |
| **Flavor** | 6,81 ±2,12 | 6,80 ±1,83 | 7,15 ±1,67 | 6,96 ±1,76 |
| **Texture** | 7,00 ±1,98 | 7,03 ±1,58 | 7,14 ±1,75 | 7,03 ±1,56 |
| **Overall assessment** | 7,20 ±1,62 | 7,13 ±1,48 | 7,53 ±1,32 | 7,10 ±1,60 |
| **Purchase intention** | 3,60^ab^ ±1,30 | 3,52^b^ ±1,22 | 3,96^a^ ±1,11 | 3,67^ab^ ±1,26 |

Data expressed as mean ± standard deviation, statistical analysis performed ANOVA followed by Tukey’s, with (p <0.05), differing letters for CVF - hydrogenated vegetable fat; CB - butter; CG - goat milk fat; CGCLA - goat milk fat with CLA.
