# Supplementary material for: Developing cookies formulated with goat cream enriched with conjugated linoleic acid
Source: PLoS One. 2019 Sep 23;14(9):e0212534. doi: 10.1371/journal.pone.0212534 (PMC6756519; doi:10.1371/journal.pone.0212534)
Supplement: S6 Table — Data expressed as mean ± standard deviation, statistical analysis performed ANOVA followed by Tukey, with different letters for (p <0.05). CVF—hydrogenated vegetable fat; CB—butter; CG—goat’s milk fat; CGCLA—goat’s milk fat with CLA. (DOCX) [file pone.0212534.s006.docx]

**Table 6. Fatty acid profile in cookies expressed in 100 mg/fat.**

| FATTY ACIDS | CVF | CB | Cg | CgCLA |
| --- | --- | --- | --- | --- |
| SATURATED |  |  |  |  |
| C4:0 | 0,01^c^ ± 0,00 | 1,29^b^ ± 0,01 | 1,61^a^± 0,02 | 1,86^a^ ± 0,01 |
| C6:0 | - | 1,31^b^ ± 0,01 | 1,87^a^ ± 0,02 | 1,54^ab^ ± 0,02 |
| C8:0 | 0,01^d^ ± 0,00 | 0,76^c^ ± 0,01 | 2,10^a^ ± 0,02 | 1,35^b^ ± 0,01 |
| C10:0 | 0,02^d^ ± 0,01 | 1,70^c^ ± 0,01 | 6,81^a^ ± 0,04 | 3,57^b^ ± 0,03 |
| C12:0 | 0,01^d^ ± 0,01 | 1,97^b^ ± 0,02 | 2,65^a^ ± 0,02 | 1,22^c^ ± 0,01 |
| C14:0 | 0,19^c^ ± 0,00 | 6,86^a^ ± 0,03 | 6,14^a^ ± 0,02 | 3,50^b^ ± 0,01 |
| C15:0 | 0,04^c^ ± 0,01 | 0,99^a^ ± 0,01 | 0,88^a^ ± 0,01 | 0,62^b^ ± 0,01 |
| C16:0 | 14,84^c^± 0,90 | 26,44^a^ ± 1,5 | 22,93^b^ ± 1,80 | 19,89^b^ ± 1,05 |
| C17:0 | 0,09^b^ ± 0,01 | 0,77^a^ ± 0,03 | 0,70^a^ ± 0,04 | 0,57^a^ ± 0,02 |
| C18:0 | 10,07^b^ ± 0,09 | 10,93^b^ ± 1,01 | 11,19^b^ ± 0,09 | 14,85^a^ ± 1,21 |
| C20:0 | 0,33^a^ ± 0,03 | 0,18^c^ ± 0,02 | 0,25^b^ ± 0,02 | 0,25^b^ ± 0,02 |
| C21:0 | 0,02 ± 0,01 | 0,01 ± 0,00 | 0,02 ± 0,01 | 0,02 ± 0,01 |
| C22:0 | 0,31^a^ ± 0,02 | 0,05 ^b^ ± 0,01 | 0,06 ^b^ ± 0,01 | 0,08 ^b^ ± 0,01 |
| C23:0 | 0,04 ± 0,01 | 0,02 ± 0,01 | 0,02 ± 0,00 | 0,02 ± 0,01 |
| C24:0 | 0,13^b^ ± 0,01 | 0,03^b^ ± 0,01 | 0,04^b^ ± 0,01 | 0,03^b^ ± 0,01 |
| TOTAL SFA | 26,11^b^ ± 0,01 | 53,29^a^ ± 4,87 | 57,26 ^a^ ± 5,40 | 49,37^a^ ± 3,80 |
| MONOUNSATURATED |  |  |  |  |
| C14:1c9 | 0,01^c^ ± 0,01 | 0,53^a^ ± 0,01 | 0,07^b^ ± 0,01 | 0,04^bc^ ± 0,01 |
| C16:1c7 | - | 0,43^b^ ± 0,05 | 0,54^ab^ ± 0,07 | 0,74^a^ ± 0,10 |
| C16:1c9 | 0,25^c^± 0,06 | 1,02^a^± 0,18 | 0,45^b^± 0,08 | 0,47^b^± 0,06 |
| C17:1c9 | 0,04^c^ ± 0,01 | 0,14^a^ ± 0,05 | 0,11^a^ ± 0,03 | 0,08^b^± 0,03 |
| C18:1t6+t8 | 2,40^a^± 0,54 | 0,18^c^± 0,05 | 0,10^c^± 0,3 | 0,34^b^± 0,10 |
| C18:1t9 | 2,23^a^ ± 0,40 | 0,13^c^ ± 0,04 | 0,10^c^± 0,03 | 0,32^b^± 0,10 |
| C18:1t10 | 5,10^a^± 0,48 | 0,21^bc^± 0,06 | 0,11^c^± 0,03 | 0,33^b^± 0,08 |
| C18:1t11 | 3,31^a^ ± 0,40 | 0,79^c^ ± 0,09 | 0,45^d^ ± 0,05 | 2,47^b^ ± 0,20 |
| C18:1t12 | 2,11^a^± 0,22 | 0,20^c^± 0,05 | 0,12^c^± 0,03 | 0,33^b^± 0,06 |
| C18:1c9 | 33,03^a^± 3,43 | 25,92^b^± 2,40 | 26,68^b^± 2,22 | 29,67^b^± 2,54 |
| C18:1t15 | 1,30^b^ ± 0,15 | 0,17^b^ ± 0,03 | 0,10^c^ ± 0,02 | 0,16^b^± 0,03 |
| C18:1c11 | 1,79^a^± 0,20 | 0,61^b^± 0,10 | 0,55^b^± 0,09 | 0,62^b^± 0,09 |
| C18:1c12 | 5,82^a^± 0,60 | 0,11^c^± 0,03 | 0,06^c^ ± 0,02 | 0,26^b^± 0,07 |
| C18:1c13 | 0,30^a^± 0,08 | 0,04^b^± 0,02 | 0,01^c^± 0,00 | 0,04^b^± 0,02 |
| C18:1t16+c14 | 0,30^a^± 0,07 | 0,18^b^± 0,05 | 0,11^b^± 0,03 | 0,26^a^± 0,04 |
| C18:1c15 | 0,29^a^± 0,06 | 0,06^b^± 0,02 | 0,02^c^± 0,01 | 0,05^bc^± 0,02 |
| C20:1 | 0,13± 0,05 | 0,06± 0,03 | 0,06± 0,04 | 0,07± 0,03 |
| TOTAL MUFA | 58,1^a^± 6,00 | 30,75^bc^± 2,80 | 29,62^c^± 2,87 | 36,20^b^± 3,50 |
| POLYUNSATURATED |  |  |  |  |
| C18:2n6 | 15,20^a^± 1,33 | 15,17^a^± 1,65 | 12,18^b^± 1,15 | 12,93^ab^±1,23 |
| C18:3n-6 | - | 0,02^b^± 0,01 | 0,20^a^± 0,04 | 0,01^b^± 0,01 |
| C18:3n3 | 0,18^b^± 0,03 | 0,25^a^± 0,05 | 0,20^ab^± 0,05 | 0,17^b^± 0,02 |
| CLAc9t11 | **-** | **0,25^b^± 0,01** | **0,21^b^ ± 0,01** | **0,95^a^ ± 0,03** |
| C20:3n-6 | 0,01± 0,00 | 0,04± 0,02 | 0,02± 0,01 | 0,02± 0,01 |
| C20:4n-6 | 0,07^b^± 0,02 | 0,13^ab^± 0,04 | 0,21^a^± 0,05 | 0,16^a^± 0,04 |
| C20:5n-3 | - | 0,01± 0,01 | 0,01± 0,00 | 0,01± 0,00 |
| C22:5n-3 | - | 0,03± 0,02 | 0,04± 0,01 | 0,03± 0,01 |
| C22:6n-3 | 0,02^b^± 0,01 | 0,03^b^ ± 0,01 | 0,04^b^ ± 0,02 | 0,12^a^ ± 0,04 |
| TOTAL PUFA | 15,48± 1,35 | 15,92± 1,40 | 13,11± 1,11 | 14,39± 1,20 |
| TRANS | 16,76^a^ ± 1,30 | 1,87^c^ ± 0,14 | 1,09^c^ ± 0,01 | 4,21^b^ ± 0,03 |
