# Supplementary material for: Developing cookies formulated with goat cream enriched with conjugated linoleic acid
Source: PLoS One. 2019 Sep 23;14(9):e0212534. doi: 10.1371/journal.pone.0212534 (PMC6756519; doi:10.1371/journal.pone.0212534)
Supplement: S1 Fig — CVF (cookie with hydrogenated vegetable fat), CB (cookie with butter), CG (cookie with goat cream) and CGCLA (cookie with enriched goat cream with CLA). (DOCX) [file pone.0212534.s007.docx]

**Figure 1.** PCA of the data for physical-chemical, physical, sensory and chromatographic parameters evaluated of the cookies: CVF (cookie with vegetable fat), CB (cookie with butter), CG (cookie with goat cream) and CGCLA (cookie with enriched goat cream with CLA) as a percentage.
